# The Association Between Lung Cancer and Sarcoidosis: A Literature Review

**DOI:** 10.7759/cureus.45508

**Published:** 2023-09-18

**Authors:** Lotanna Umeano, Hari Priya Pujari, Syed Muhammad Zain Jamil Nasiri, Anusha Parisapogu, Anuj Shah, Jamal Montaser, Lubna Mohammed

**Affiliations:** 1 Internal Medicine, California Institute of Behavioral Neurosciences and Psychology, Fairfield, USA; 2 Diagnostic Radiology, California Institute of Behavioral Neurosciences and Psychology, Fairfield, USA; 3 Infectious Disease, California Institute of Behavioral Neurosciences and Psychology, Fairfield, USA; 4 Infectious Disease, Mayo Clinic, Rochester, USA; 5 Psychiatry, California Institute of Behavioral Neurosciences and Psychology, Fairfield, USA

**Keywords:** granuloma, sarcoid, sarcoidosis, lung neoplasm, lung cancer

## Abstract

Lung cancer is responsible for a significant number of cancer-related deaths worldwide. While various factors can lead to lung cancer, such as tobacco smoking, this article focuses on the relationship between sarcoidosis, a multisystem granulomatous disorder, and lung neoplasm. To investigate this association, the authors conducted a literature search using relevant keywords. The analysis of these reports concluded that while Sarcoidosis and lung cancer together is rare, it is possible. The presenting symptoms, age, gender, and diagnostic procedures of each case should be evaluated, and appropriate diagnostic procedures should be carried out to determine the appropriate treatment for each patient. Clinicians need to be aware of the possibility of these two diseases co-occurring, as they can impact the management of the patient's condition, whether it is curative or palliative. It is essential to rule out metastatic cancer in individuals with sarcoidosis-like clinical and radiographic features.

## Introduction and background

Lung cancer is a severe concern today, as it has become the leading cause of cancer-related deaths for both men and women worldwide. Its insidious onset, early metastasis, and poor prognosis make it all the more concerning [1]. It is essential to be aware of the risk factors of lung cancer and take steps to reduce our exposure to them. Interestingly other factors besides tobacco can contribute to lung cancer. Studies have shown that environmental exposure to cigarette smoke, radon, occupational carcinogens, pre-existing nonmalignant lung illness, and genetic variables can all increase an individual's risk of developing lung cancer. It is essential to be mindful of these potential risk factors and take preventative measures to minimize our exposure to them [2]. According to the histological pattern, lung cancer can be divided into two groups: small-cell lung carcinomas (SCLC) and non-small-cell lung carcinomas (NSCLC), with varying prognoses for each group [3].

Sarcoidosis affects multiple body areas, including the lungs, lymph nodes, liver, eyes, and skin. The disease is systemic and characterized by the formation of multiple granulomas of unknown origin [4]. However, according to some studies, young men occupationally exposed to silica dust may face an increased risk of developing sarcoidosis [5]. It is suggested that occupational exposure to inorganic dust, such as silica/silicates and metals, may increase the risk of developing sarcoidosis [6]. Sarcoidosis is becoming more prevalent globally. One study found the incidence of sarcoidosis in the United States to be between 7.6 and 8.8 per 100,000 population per year [7].

Individuals with sarcoidosis must receive proper medical care and monitoring to manage potential complications and improve their overall health outcomes. Respiratory failure, pulmonary fibrosis, central nervous system involvement, and cardiac damage are severe risks associated with sarcoidosis, which can be life-threatening if not adequately addressed [7]. It is essential to consider sarcoidosis as a potential diagnosis for any young or middle-aged adult who presents with unexplained cough, shortness of breath, or constitutional symptoms, particularly among blacks or Scandinavians. To diagnose sarcoidosis, doctors rely on three criteria: a clinical and radiologic presentation consistent with the disease, pathologic evidence of non-caseating granulomas, and excluding other diseases with similar findings, such as infections or malignancy [8]. A clinically distinct phenotype of sarcoidosis, Lofgren syndrome, typically presents acutely in younger patients with acute onset erythema nodosum (EN), bilateral hilar lymphadenopathy, fever, and migratory polyarthritis, and without granulomatous skin involvement [9].

Proper diagnosis is crucial to ensure that individuals with sarcoidosis receive the appropriate medical care and monitoring to manage potential complications and improve their overall health outcomes. Table 1 describes the radiographic features of different stages of sarcoidosis.

**Table 1:** Features and frequency of pulmonary sarcoidosis stages

Stage	Radiographic features	Frequency at presentation (%)
I	Mediastinal and hilar adenopathy (usually bilateral) without pulmonary infiltrates	40-50
II	Mediastinal and hilar adenopathy (usually bilateral) with pulmonary infiltrates	30-40
III	Pulmonary infiltrates without adenopathy (adenopathy already regresses)	15-20
IV	Pulmonary fibrosis with volume loss. No adenopathy	2-5

Based on available data, the outlook for individuals with pulmonary sarcoidosis is positive. Up to 80% of patients with stage I disease have been observed to experience spontaneous regression of radiographic abnormalities. Additionally, the rate of development of chronic respiratory impairment is relatively low, affecting less than 5% of patients over 10 years [7]. It is important to note that the prognosis for those with more advanced stages at diagnosis is less favorable. Unfortunately, spontaneous regression of radiographic abnormalities is only seen in about one-third of patients with stage II and III disease [5,7]. Patients with stage III and IV disease have a fivefold increased risk of chronic respiratory impairment compared to those with stage I [7].

Corticosteroid medications are a commonly prescribed treatment for individuals with severe symptoms and advancing pulmonary disease or significant extrapulmonary illness caused by sarcoidosis. In some cases, immunosuppressive treatment may also be necessary [3].

This study focuses on rare cases reported and research articles published globally in the last 10 years to examine the relationship between sarcoidosis and lung cancer and determine whether there is a relationship between the two. It also offers guidance to doctors on how to manage such uncommon encounters.

## Review

Even though there have been numerous documented associations, the relationship between lung cancer and sarcoidosis is still unclear. The authors break down the discussion session into theories, the diagnostic challenges, and the inflammatory responses related to sarcoidosis that may lead to neoplasm.

Theories

Cancer seems more common in sarcoidosis patients, including malignancies of the liver, stomach, small intestine, lung, and skin, as well as non-Hodgkin's lymphoma and leukemia. Chronic inflammation is associated with a high risk of malignant lymphoma or cancer in the affected tissue [10]. Theoretically, this could also apply to sarcoidosis, which most often involves intrathoracic organs, the liver, and the skin. Compared to other chronic conditions, including diabetes [11], inflammatory bowel disease [12], and rheumatoid arthritis [13], the estimated overall relative risk for cancer in patients with sarcoidosis is equivalent to or slightly greater [10].

Epidemiological studies show that at least 25% of patients with sarcoidosis and neoplasm may have an etiological relationship [14]. Numerous studies have examined the link between sarcoidosis and lung cancer; one of these is the study by Brincker and Wilbek, which examined 2,544 patients and discovered that those with sarcoidosis had a threefold increased risk of developing lung cancer [15]. However, it is still unclear how exactly sarcoidosis and lung cancer are related [16]. The causes of this association were hypothesized by the Sakula study in 1963, which identified three possibilities: (1) sarcoidosis precedes lung cancer and is somehow connected to, and may even potentially trigger, the malignant transformation due to the post-inflammatory scar tissue; (2) sarcoidosis may develop as a consequence of lung cancer; and (3) the onset of sarcoidosis precedes the onset of lung cancer, and the incidence of lung cancer and sarcoidosis is coincidental [17]. According to another study by Yamasawa et al., there are four hypotheses regarding the cause-and-effect relationship between sarcoidosis and lung cancer: (1) the two diseases coexist incidentally;(2) the cell-mediated immune abnormalities induced by sarcoidosis are involved in the onset of lung cancer;( 3) sarcoidosis causes lung cancer to proliferate in the fibrous tissue, and ( 4) the onset of sarcoidosis is caused by an immunohistological reaction such as sarcoid reactions, which respond to malignant tumors [18]. Sarcoid reactions refer to the development of non-caseating epithelioid cell granulomas in patients who do not fulfill the criteria for systemic sarcoidosis. Another theory claims that sarcoidosis is an immune reaction to tumor antigens. This hypothesis is supported by observations that solid and lymphohematogenous tumors can produce a sarcoidosis-like systemic granulomatous response [19].

In a study conducted in Japan from 1980 to 2007, three cases of sarcoidosis and lung cancer were noted. In one case, the patient developed lung cancer 16 years after being diagnosed with sarcoidosis. In another case, the patient had two different lung cancers, occurring 10 and 18 years after the diagnosis of sarcoidosis. In the third case, both illnesses were detected simultaneously. However, It can be challenging to ascertain whether the coexistence of non-caseating epithelioid cell granulomas with lung cancer in simultaneously detected cases represents a lung cancer reaction or genuine systemic sarcoidosis. These three cases were shown to have smoking indexes of 300,720 and 70, respectively [20].

Ungprasert et al. discovered a higher incidence of malignancy among patients with sarcoidosis than those without sarcoidosis. However, it is essential to note that this relative risk was insignificant once detection bias was accounted for [21]. In another meta-analysis, lung cancer's relative risk rises within four years following a sarcoidosis diagnosis [22]. Furthermore, in a case report, SCLC diagnosis was made two to 12 years after the sarcoidosis diagnosis. The median time between a sarcoidosis diagnosis and the subsequent development of SCLC was 5.6 years. It is interesting to note that Hatakeyama et al. documented a case of a patient who had spontaneously remitted from sarcoidosis before being given a SCLC diagnosis two years later [16]. Although this relationship has been studied for many years, the data is still unconvincing because of conflicting findings and insufficiently large cohorts (fewer than 500 cases in 65% of studies) [23].

Diagnostic challenges and differential diagnosis

A non-caseating granuloma can help confirm a diagnosis of sarcoidosis, especially in optimal clinical settings [24]. In addition to confirming a diagnosis of sarcoidosis, it has been observed that non-caseating granulomas can also exist in correlation with a malignancy, known as a sarcoid-like response. Sarcoid reactions have occasionally been noted in the lymph nodes, draining the malignancy in individuals with malignant disease, as stated in the fourth hypothesis by Yamasawa et al. They may also develop in the stroma, the tumor's primary organ, or distant tissue sites such as the spleen, bone marrow, and skin [14].

Sarcoid-like reactions are autoimmune responses that resemble the sarcoidosis symptoms. Different etiologies, including infections most frequently, might cause this reaction. Additionally linked to sarcoid-like reactions are cancer, medicines, and other autoimmune responses [25]. These triggers activate antigen-presenting cells, including alveolar macrophages and interstitial dendritic cells, by mimicking the actions of exogenous antigens. They then trigger the recruitment and activation of T and B lymphocytes, which results in an IFN-mediated hyperimmune state in the body [26].

Cases with concurrent sarcoidosis and malignant disease may help illuminate the causal relationship between them, even though the distinction between sarcoidosis and sarcoid responses in their mechanisms of development is still unclear [20]. These granulomas may be found near the cancer's location or in the surrounding lymph nodes. Therefore, the existence of granulomas does not always rule out malignancy since non-caseating granulomas have been seen in some cases of SCLC and because mediastinal lymph nodes are involved in both sarcoidosis and lung cancer [27]. When lung cancer and non-caseating epithelioid cell granulomas are seen together, it might be challenging to determine whether they represent signs of a sarcoid reaction or definitive systemic sarcoidosis [20].

A popular diagnostic tool for detecting increased metabolic activity, fludeoxyglucose-positron emission tomography (FDG-PET), has a 96% sensitivity and a 79% specificity for lung cancer. The standardized uptake value (SUV), used to quantify a tumor's metabolic activity, is highly predictive of malignancy when it is more significant than 2.5. This test, however, may yield false-positive results in granulomatous diseases like sarcoidosis [28]. The lymph nodes in a case report of a patient with a history of sarcoidosis who was diagnosed with SCLC had SUVs as high as 4.6, which further complicated the clinical picture because it was impossible to determine whether this was secondary to sarcoidosis or lung cancer [29].

Even though the likelihood of sarcoidosis and lung cancer coexisting is extremely low (less than 1%) of the time, it is more frequently seen in squamous cell lung cancers [29]. When diagnosing sarcoidosis, it is important to consider all possible causes for concerning symptoms, including lung cancer [20]. A lung biopsy from any nodular lesion in a sarcoidosis patient is essential for the differential diagnosis and initiating treatment as soon as possible [30]. Doing tissue biopsy from intrathoracic lymphadenopathies has been common practice using endobronchial ultrasonography (EBUS) guidance during transbronchial needle aspiration (TBNA). However, because some lymph nodes are challenging to access with EBUS, it may be challenging to biopsy all intrathoracic lymph nodes using EBUS-guided TBNA.

According to Koda et al., some textural traits have been shown to be able to distinguish SCLC from sarcoidosis in patients with mediastinal lymphadenopathy on CT. The combination of EBUS elastographic fat-to-lesion strain ratio and textural feature grey-level run length matrix with high gray-level run emphasis yielded nearly excellent diagnostic capability for discriminating between these two diseases. Therefore, enlarged mediastinal lymph nodes with CT-based texture analysis may help distinguish between sarcoidosis and other diseases that cause mediastinal lymphadenopathy, such as malignant lymphoma and metastatic lymph nodes associated with NSCLC [31].

Pulmonary sarcoidosis has frequently been misdiagnosed as lung cancer, according to reports. In a rare instance, pulmonary sarcoidosis was mistakenly diagnosed as progressing lung cancer, delaying the detection of the disease [32]. The misdiagnosis of sarcoidosis in lung cancer patients may explain the elevated risk during the first years of follow-up. However, considering the poor prognosis for lung cancer, such misdiagnosis is an unlikely explanation for the increased risk, for instance, after five to nine years of follow-up. Although the preclinical stage of lung cancer may last for a long time, it is improbable that it will last longer than, say, five years from the presentation with (bilateral) hilar enlargement that was initially misdiagnosed as sarcoidosis. However, we cannot entirely rule out the possibility [10].

Medication safety margin in immunotherapy in alternative theories contends that lung cancer chemotherapy, tyrosine kinase inhibitors, or immunological checkpoint inhibitors like ipilimumab and nivolumab that trigger local sarcoid responses may be the cause of sarcoidosis [3]. In just four months after beginning chemotherapy (carboplatin/pemetrexed) and pembrolizumab combination therapy, Fakhri et al. found pulmonary sarcoidosis in a patient with NSCLC, indicating that immune checkpoint inhibitors may cause immuno-induced sarcoidosis, which may be mistaken for lung cancer progression [33]. Leishmaniasis, tuberculosis, and coccidioidomycosis, among other microbial infections, have been linked to sarcoid-like reactions in other investigations; as a result, biopsies from two nonadjacent areas are indicated in patients with atypical sarcoidosis in order to rule out an active tumor or infection [27].

Inflammatory response to sarcoidosis

There are conflicting views on the link between sarcoidosis and lung cancer. Some studies suggest that people with sarcoidosis may be at a higher risk of developing lung cancer and experiencing mortality. In contrast, others indicate that the incidence is not higher than expected. Nevertheless, the persistent inflammation and scarring that come with sarcoidosis and abnormalities in cell-mediated immunity could potentially be a factor in triggering cancer development [16].

Active sarcoidosis is characterized by an enhanced local expression of T helper 1 (Th1) and T helper 17 (Th17) chemokines and cytokines like IFN-γ, TNF-α, IL-17A, and IL-22. In various chronic, autoimmune, inflammatory diseases, such as sarcoidosis, the percentage of IL-17A+/IFN-γ+ double-producing Th-cells increases in peripheral blood and is related to high disease activity [27-26]. Furthermore, in these pathological conditions, a dysfunctional response of regulatory T-cells (Tregs) has been described that is characterized by an impaired immunosuppressive function [34].

Interestingly, during sarcoidosis, the expression of cytotoxic T-lymphocyte antigen 4 (CTLA-4) is decreased, while the expression of programmed death-1 (PD-1) is increased in Th17-cells in the mediastinal lymph nodes [18]. Th-cells include CTLA-4, which exerts a limiting influence on subsequent T-cell responses. A decreased CTLA-4 level thus maintains inflammatory responses. Similarly, T-cell activation results in the expression of PD-1, which limits inflammatory responses. Tumor cells contain its ligand, PD-1L, which causes T-cells to express more PD-1. As a result, the activation of tumor antigen-specific T-lymphocytes is inhibited [35]. As a result, the adaptive immune response in sarcoidosis and metastasis patients maintains sarcoidosis-related inflammation because of decreased anti-inflammatory CTLA-4 expression while restricting tumor-specific T-cell activation, indicated by an increased PD-1 expression, which enables tumor escape from the immune system and metastasis [35-36]. Thus, increased PD-1 expression on T-cells in lymph nodes associated with sarcoidosis may be a potential indicator of the development of cancer and metastases from sarcoidosis [35].

Furthermore, S100A8/A9 (belongs to the Ca2 + binding S100 protein family) levels in serum are higher in sarcoidosis patients, and monocytes and multinuclear giant cells express S100A8/A9 more cytoplasmically in granulomas. We might surmise that the inflammation associated with sarcoidosis, which is characterized by the expression of IFN, IL6, IL23, and S100A8/A9, shares a milieu with malignancies, which are distinguished by an elevated accumulation and immunosuppressive role of myeloid-derived suppressor cells (MDSC). As a result, sarcoidosis can encourage metastasis by causing cell subsets that promote tumors and regulate the immune system [36]. More research is needed to confirm the impact of MDSC on sarcoidosis and the cellular immune response to the relationship between sarcoidosis and metastasis.

Limitations

Firstly, many of the studies are retrospective, which may introduce bias and limit the ability to establish causality. Additionally, diagnosing sarcoidosis may be complex and subject to inter-observer variability, leading to misclassification and underestimation of the true prevalence of sarcoidosis in lung cancer patients. Furthermore, the heterogeneity of sarcoidosis in terms of clinical presentation and disease course may make it challenging to identify specific subtypes more strongly associated with lung cancer. Finally, the studies included in this review varied widely in terms of sample size, study design, and patient population, which may limit the generalizability of the findings. These studies cited in the article have not demonstrated a clear control adjustment for other variables, especially the ones that cause lung cancer including smoking, environmental exposure, etc. Overall, while the association between lung cancer and sarcoidosis is an essential area of research, further studies are needed to understand better the underlying mechanisms and potential clinical implications of this relationship.

## Conclusions

When dealing with a potential case of sarcoidosis, it is crucial to remember that it can mimic various other diseases, including infectious, neoplastic, and granulomatous diseases. Furthermore, individuals with sarcoidosis may be more susceptible to lung cancer. This is consistent with the elevated risk of cancer reported in other inflammatory respiratory diseases, and rather than sarcoidosis itself, the risk may be mediated by the accompanying cell regeneration. Therefore, conducting a thorough assessment and maintaining a diverse differential diagnosis is vital. Even though sarcoidosis and lung cancer seldom coexist, doctors should investigate the possibility of lung cancer in sarcoidosis patients who do not respond to early corticosteroid treatment and obtain a biopsy to establish pathological findings from any suspected lung lesion.

While there is no evidence of a link between sarcoidosis and lung adenocarcinoma, physicians should ensure no lesion is overlooked in individuals without a biopsy. This structured approach can improve the patient's outcome and prognosis while detecting malignancy earlier. Given the diagnostic conundrum of not being able to differentiate sarcoidosis and lung cancer, it is better to treat the nodal involvement as SCLC metastasis because failure to do so might result in relapse and jeopardize survival. Lastly, more cohort studies are required to enhance the body of literature already in existence. It may lead to the discovery of answers to the link between lung cancer and sarcoidosis.
